# Exploring nurse managers’ perception of using the RAFAELA system as a management tool in a Norwegian hospital setting

**DOI:** 10.1002/nop2.115

**Published:** 2017-12-14

**Authors:** Bodil Mørk Lillehol, Kjersti Lønning, Marit Helen Andersen

**Affiliations:** ^1^ Division of Surgery Inflammatory Diseases and Transplantation Oslo University Hospital Nydalen Norway

**Keywords:** focus group interviews, medical and surgical hospital units, nurse managers’ perceptions, patient classification system

## Abstract

**Aim:**

The aim of the study, being part of a Norwegian evaluation project of the RAFAELA system, was to explore nurse managers’ perception of the RAFAELA system as a management tool in a Norwegian hospital setting.

**Design:**

We applied an explorative qualitative design using focus group interviews.

**Methods:**

Two focus group interviews were performed with 12 nurses in different management positions during autumn 2013. The principles of qualitative content analysis were used for analysing data.

**Results:**

Three themes emerged. The informants experienced the RAFAELA system to be a basis for a precise and common langue. Furthermore, the informants considered it to be a system defining quality standards of nursing care. Finally, the RAFAELA system provided daily documentation of nursing intensity and thus was considered an important management tool for balancing patient needs with appropriate staff.

## 1 INTRODUCTION

Advancements in medicine have led to more and more complex patient needs. Nurses’ contribution to the treatment and care of patients, which can be measured through nursing intensity (NI), defined as patients’ need for care and the nursing interventions needed to ensure good care (Rafferty et al., 2007), has a direct impact on quality and outcome (Aiken et al., 2014; Fagerström, Lønning, & Andersen, 2014; Keogh, 2013; McHugh, Berez, & Small, 2013; Needleman et al., 2011).

Throughout the world, more cost‐effective health services are sought. At the same time, an important goal for nursing is that patients have the right to high quality care and treatment. (Andreasson, Eriksson, & Dellve, 2015; Brown, Donaldson, Burnes Bolton, & Aydin, 2010; Fagerström & Rauhala, 2007; Lang, Hodge, Olson, Romano, & Kravitz, 2004; Ruland & Ravn, 2003; Spence et al., 2006).

The use of validated nursing tools for balancing true patient needs with appropriate nursing resources has become important. The Finnish patient classification system RAFAELA, including the Oulu Patient Classification instrument (OPCq), was designed to measure NI and nursing staff allocation (Rauhala & Fagerström, 2004). In 2011, we started a broad evaluation project of the RAFAELA system as used at Oslo University Hospital in Norway (OUS) to test the international relevance of the RAFAELA system and to investigate if the system was able to provide valid data as used in a Norwegian hospital setting. We here report from the fourth sub‐study aiming to explore nurse managers’ perception of the RAFAELA system as a management tool in a Norwegian hospital setting.

### 1.1 Background

The RAFAELA system was designed in Finland during the 1990s to measure nursing intensity and nursing staff allocation in medical and surgical wards (Frilund & Fagerström, 2009; Pusa, 2007; Rauhala & Fagerström, 2004). The system consists of a three‐part data collection system. The first component (the OPCq) measures daily NI by determining the individual caring needs of the patients through six nursing domains: (i) planning and coordination of care; (ii) breathing, blood circulation and symptoms of disease; (iii) nutrition and medication; (iv) personal hygiene and excretion; (v) activity/movement; and (vi) teaching, guidance and follow‐up. The second component provides daily registration of actual nursing resources for direct care (N), whereas the third component represents Professional Assessment of Optimal Nursing Care Intensity Level (PAONCIL) for each unit. The latter can be described as the nurses’ assessments of the sufficiency of resources in relation to the actual NI of patients during a shift, defined at each unit. The workload is expressed in OPCq/N and is then compared with the unit's optimal standard for care intensity level (Fagerström, Rainio, Rauhala, & Nojonen, 2000; Rauhala & Fagerström, 2004). The following example provided by Fagerström et al. demonstrates how workload is expressed using the RAFAELA system: “The NI can vary for each subarea from 1–4 points. The points are added up, giving a range of 6–24 NI points per patient. The total sum of NI points for all patients in the unit is then calculated, for example, 240 points. Then, the total sum of NI points for a unit is divided by the total number of nurses who had nursed the patients in the unit during that calendar day (e.g. 10 nurses). As seen in our example, the actual NI level would be 24 NI points per nurse” (Fagerström & Vainikainen, 2014, p2). The actual NI level from this example—24 NI—indicates rather high nurse intensity this day.

The RAFAELA system is widely used in Finland today (Fagerström et al., 2014). The last decade it has also been introduced to several countries in Northern Europe, both in an established manner (e.g. Iceland) and in an evaluating perspective (e.g. the Netherlands). The work presented here is one of four reports outgoing from the Norwegian evaluation project of the RAFAELA system. In the first sub‐study, evidence was provided for sufficient reliability and validity of OPCq as used at OUS (Andersen, Lønning, & Fagerström, 2014). In sub‐study 2, we explored hospital managers’ experiences of the RAFAELA system using an interdisciplinary perspective, including all management levels of the division (Hustad, Hellesø, & Andersen, 2015). Differences in nursing intensity and costs in two different patient groups were demonstrated in sub‐study 3 (Andersen, Lønning, Bjørnelv, & Fagerstrom, 2016). As nurse managers are responsible for systematic work on high quality of patient care and the RAFAELA system was developed as a management tool for nurses, we set out to study how nurses in management positions regarded the system in sub‐study 4.

## 2 THE STUDY

### 2.1 Design

A qualitative, explorative research design was chosen to generate a deeper understanding of the nurse managers’ perception of using the RAFAELA system as a management tool. We used focus group interviews for data collection to stimulate the participants to discuss their experiences of the system through broad discussions. The focus group dynamics can generate new thinking about a topic which may result in a more in‐depth discussion as compared with individual interviews. Hence, the presence of several informants participating in open, spontaneous conversations may enhance richness of data (Krueger & Casey, 2009; Rice & Ezzy, 1999).

### 2.2 Setting and sample

Twelve nurse managers from six different medical and surgical clinical units participated in the study. All units at the hospital having experience with using the RAFAELA system were represented in the study. The informants had between 2–8 years of experience working in nurse management positions at section—or unit level. The first author (BML) recruited informants who had at least 6 months experience with use of the RAFAELA system and who had a manager position. Before the interviews, the participants were given oral and written information about the study and about the confidential nature of all data collected. Background characteristics of study informants are showed in Table 1.

**Table 1:** Background characteristics of study informants (*n *=* *12)

Informants	Units	Nurse management level	Years of manager experience
1	Unit of Rheumatology and Infection	Assistant section manager	<5
2	Unit of Dermatology	Assistant unit manager	<5
3	Unit of Gastro/Urology Surgery	Unit manager	<5
4	Unit of Transplant Surgery	Assistant unit manager	>5
5	Unit of Rheumatology and Infection	Section manager	>5
6	Unit of Gastro/Urology Surgery	Section manager	<5
7	Unit of Transplant Surgery	Unit manager	>5
8	Unit of Rheumatology and Infection	Assistant unit manager	>5
9	Unit of Dermatology	Assistant unit manager	>5
10	Unit of Gastro/Nephrology Medicine	Unit manager	<5
11	Unit of Transplant Surgery	Assistant unit manager	>5
12	Unit of Gastro/Urology Surgery	Assistant unit manager	<5

### 2.3 Data collection

Two focus group interviews were conducted during autumn 2013. An interview guide was developed to ensure that important topics were covered. The topics were based on previous research about the RAFAELA system (Fagerström et al., 2000; Rauhala & Fagerström, 2004), the researchers’ experiences of implementing the system at the OUS and local knowledge about patient groups, staffing and organizational matters. The interviews started with a short briefing about the purpose of the study. The interview questions were formulated to invite participants to reflect together. Follow‐up questions were asked to enable exploration of issues and obtain detailed descriptions, like “How will you describe..?” or “What is your experience of..”? After the interview, the participants were given the opportunity to contact the researchers to discuss their experience of the interview situation, or to add other experiences BML performed the interviews, whereas a moderator (KL) insured that predetermined issues were covered during the interviews. Reports were coded anonymously. Both interviews were audio‐recorded and verbatim transcribed by BML.

### 2.4 Analysis

The interviews were analysed using Kvale & Brinkman's method for content analysis. We performed an inductive analysis process to generate meanings from the raw data to identify patterns and relationships. First, each interview was read carefully to get an overall impression of the data material. Then the text was divided into meaning units, being one or more sentences from the raw data marked in the coding process, comprising information about the research question and referring to the smallest unit coded. Third, the theme that dominated a natural meaning was stated as simply as possible. The fourth step consisted of analysing the meaning units in terms of the specific purpose of the study. In the fifth step, the essential themes of the entire interview were tied together in a descriptive text (Kvale & Brinkmann, 2009).

#### 2.4.1 Trustworthiness

Three researchers performed the study (BML, KL and MHA). The research question and the interview guide were based on literature reviews and the researchers’ experience in the field. The focus group interviews were performed by two researchers, one of them interviewing (BML) and the other adding relevant follow‐up questions and insure integrity of the participants (KL). Data analysis started with the researchers reading the transcripts separately. Then two members of the research group (BML, MHA) met regularly to define meaning units from the data, make categories and propose preliminary themes. Then the third researcher (KL) joined the group to debate different ways of interpreting, categorizing and organizing the data.

### 2.5 Ethics

The study was assessed and approved by the internal Data Protection Officer at OUS (#2010/27572). Informed consent to participate was obtained from all participants. In this study, the anonymity of the informants might be threatened because the informants could be easily recognizable. We addressed confidentiality of the informants during all phases of the study, including dissemination of research results. If the informant statements contained references to persons or places, or specific occasions difficult to capture, name of persons and units were removed from the data set, or details in data were modified, however with minor implications for the results.

The research group included one master student (BML) and two experienced researchers in qualitative research methodology (KL and MHA) to strengthen the scientific perspective and ensure optimal ethical considerations throughout the different study phases. In the hospital setting, MHA was the senior research nurse in the department and KL was a clinical coordinator, none of them part of the leader group. BML was head nurse at one of the participating units.

The RAFAELA system is owned by the Association of Finnish Local and Regional Authorities and its use is managed by the FCG (Finnish Consulting Group Ltd). The actual study was initiated by OUS, hence the last author of this paper, MHA, made the first contact with FCG. The license to use the system was acquired through a standard agreement between OUS and FCG.

## 3 RESULTS

Three main themes emerged from the data analysis: (i) Basis for a precise, common language; (ii) A system defining quality standards of nursing care; and (iii) An important management tool for balancing patient needs with appropriate staff.

The informants experienced the RAFAELA system to be a useful basis for a precise and common language in nursing as the system aided to focus on nursing essentials and develop the profession. Also, the RAFAELA system improved communication about patient needs and quality of nursing care in the wards. Finally, the system provided daily documentation of nursing intensity and thus was considered an important management tool for balancing patient needs with appropriate staff.

### 3.1 Basis for a precise, common language

The precise terms in the RAFAELA system are meant to provide a basis for a clear common language to improve communication about patient needs (expressed by patients) with patients and among nurses. The informants agreed that RAFAELA had helped them to focus on how to define nursing and the profession as such. The six parts of the OPCq instrument ((i) planning and coordination of care, (ii) breathing, blood circulation and symptoms of disease, (iii) nutrition and medication, (iv) personal hygiene and excretion, (v) activity/movement, and (vi) teaching, guidance and follow‐up) helped nurses focus on the essentials in their profession. Repeated use of the system at fixed times systematized the communication process in a helpful way:

I think it increases awareness. Maybe it even helps us produce better documentation. We have to write in a precise and detailed way. It is important when others read what we have written and classify patients on the basis of it.

Nurses are trained to meet patient needs. How do we know what they need? I find that RAFAELA helps us to see their individual needs. When the patient is categorized we actually know what we're doing and how we spend our time.

The precise terms used, the nature of the information requested and regular use of the three RAFAELA components systematized and increased the level of communication among nurses within and across organizational levels.

### 3.2 A system defining quality standards of nursing care

The process of using the RAFAELA terms and components to document actual NI and discuss optimal NI increased awareness, reflection and engagement about these issues among the nurses. It also improved communication between them on how they spent their time in relation to patient needs and how they defined quality care and prioritized on that basis. All of this was helpful for developing common values, quality standards and goals:

RAFAELA raises the nurses’ awareness, I'm sure about that. RAFAELA shows what we do and how we do it. We also had training of using the system and RAFAELA inspired us to discuss what type of nursing we provide. We discussed a lot about how we wanted to do things.

RAFAELA does affect the quality of the nursing. We now speak more about what kind of nursing we wish to provide and how we want to do things in our unit.

It's about the patients’ time. And it's about respecting this time.We had many discussions about our profession while introducing RAFAELA and we have continued that. It is very useful to participate in such discussions about what we actually do, how we spend our time at work as nurses, how we prioritize and why. It has helped us to increase our awareness about how we spend our time at work.

### 3.3 Improved NI documentation, resource allocation and possibility of tailored training

The RAFAELA system provided daily documentation of nursing intensity and thus was considered an important management tool for balancing patient needs with appropriate staff.

The RAFAELA reports—produced through the process outlined above—provided a regularly updated self‐developed evidence‐base for planning of NI. The reports function as tools for systematizing expectations and results within and across organizational levels. They are important for building arguments during the budget process, provide overviews of hospitalized patients at various times and document that a great variation in workload creates unpredictable peaks and troughs (variation in acute cases) that should be met with extra help. The reports help to plan a better work shift and document a need for new positions. They also help nurses have an impact on their own work situation, map the need for training and present the departments’ activities more accurately and professionally during recruitment. Nursing resources were allocated more appropriately as a consequence of the reports. Good resource allocation was said to have the potential to strengthen the relationship between leaders and collaborators:

RAFAELA reports become an increasingly important tool for us and we use them in the budget process.

I print out the reports each month, hang them on the billboard in our common room and then I refer to these reports at the personnel meetings.

It means that we can give nurses who are interviewed (for recruiting purposes) a good picture of the type of patients we have and the type of tasks one can expect in this exact ward. It also helps us to tailor training of new nurses.

Our study demonstrated some challenges related to the RAFAELA system. The informants considered the system to be time‐consuming and especially during the implementation phase. However, as they saw it, using the system most likely would become a natural part of everyday work.

RAFAELA is time‐consuming and it has been a challenge to remember to classify all patients. I see that we have to follow up and control that patients are classified every day even after a long time. We had not anticipated that.

We have to use RAFAELA for a while before we understand what kind of tool it is, which is natural. But, it's just a question about time before classification becomes a natural part of our everyday work.

The informants also experienced that the RAFAELA system did not take into account the employees’ level of competence and experience: i.e. in the overview of resources a nurse with many years of experience and a high level of competence is registered in the same way as a newly educated nurse. As a result, this could imply that nursing resources in the wards did not reflect the actual situation and that reports could be misinterpreted.

## 4 DISCUSSION

The nurse managers found the RAFAELA system to provide an important basis for a clear common language that strengthened communication about patient needs. Daily classification of patient needs helped the nurses keeping their focus where they meant it should be. RAFAELA clearly helped to align the nursing profession with the goals of the hospital, among others to base their activity on patient needs. Daily classification of patient needs helped the nurses to keep their focus where they meant it should be. The lack of a standardized language in nursing is considered an important obstacle for good quality care (Fasoli & Haddock, 2010) and using a common reference frame in a clinical setting may lead to better communication among nurses and other healthcare providers, increased visibility of nursing interventions and improved patient care (Rutherford, 2008). The informants’ positive attitude to the RAFAELA system may also be explained by organizational changes in Norwegian hospitals the last decades. As chief nurses have been replaced by medical division managers, many nurse managers experience loss of control concerning the context of nursing practice. Using the RAFAELA system allows for balancing patient needs to suitable staffing and may in such strengthen nurse managers’ ability to predict and ensure quality care.

A prerequisite for purposeful application of RAFAELA is involvement of the entire nursing staff in discussions about all the three RAFAELA components (Fagerström et al., 2014). Involvement of nurses in the process of determining optimal NI was perceived as a very valuable and motivating aspect of using RAFAELA according to the informants. The required discussions about patient needs, actual NI and optimal NI increased awareness, reflection and engagement among the nurses, regarded as essential for improving quality of nursing in clinical hospital wards. Previous reports support the importance of involving employees directly in discussions to improve nursing care processes and nursing quality (Andreasson et al., 2015; Needleman & Hassmiller, 2009).

The implementation of the system also raised the discussion on which values their actual and ideal prioritization were based on and whether there was consensus in the staff. This is important as it is well known that the opposite situation, lack of clarity about goals, often leads to divergent approaches and slow progress in performance improvement (Porter, 2010). Managers who use the RAFAELA system are prompted to conduct relational management by listening to collaborators and inviting them to influence their own working situation. That type of collaboration tends to create positive feelings between leaders and subordinates and increases the likelihood of reaching goals. RAFAELA is probably especially suitable in a Nordic culture where employees expect to be involved in processes and decisions, rather than just being told how to do things. In a study of the impact of hospital care environment on patient mortality and nurse outcomes (Aiken et al., 2011) concluded among others that a good work environment with an inherent possibility of developing the profession, is essential to achieve high quality care.

Another central finding in our study was that the informants regarded RAFAELA as a useful tool for daily resource allocation and strategic planning. The RAFAELA reports provided the managers with daily documentation of nursing intensity. Thus, the system was considered an important management tool for balancing patient needs with appropriate staff. This finding supports the main idea of the RAFAELA system which is to uphold staffing level in accordance with patients’ care needs (Fagerström et al., 2014). In a study of van Oostveen et al., it was found that using an instrument explaining patient care needs and costs of care helped healthcare professionals and managers to balance actual patient needs with appropriate nursing resources (Van Oostveen, Ubbink, Huis in het Veld, Bakker, & Vermeulen, 2014). In a report of Baernholdt and Cottingham (2011), the authors recommend in‐hospital structures of information systems as useful management tools.

Our findings also captured negative experiences of using the system. Several informants stated that the high amount of follow‐up of the classifications and controls needed to check numbers of patients classified had been unexpected; others stated that it required time to thoroughly understand the nature of the system. It is easy to understand these statements as we implemented a quite complex system into busy hospital wards. Previous research studying similar processes support that implementing patient classification system is time‐consuming (Fasoli & Haddock, 2010; Harper & McCully, 2007). Integrating the RAFAELA system into the hospital's health information system is crucial when implementing the system into regular practice. This will probably lead to a higher amount of patients being classified, which is in general important for an effective and secure use of the RAFAELA system. For the future such integration also may allow for monitoring nursing quality indicators that focus on nursing‐sensitive patient outcomes.

A solution concerning time spent to educate the staff could be to use web‐based introduction programmes. When it comes to the managers’ notion that the RAFAELA system did not take into account the employees’ level of competence and experience, this is a serious limitation of the system. According to Fagerström, integrating nurse competence is regarding essential for further development of the RAFAELA system (Fagerström et al., 2014).

### 4.1 Study limitations

Though we tried to enhance trustworthiness throughout the study by different strategies, some study limitations should be mentioned. The study participants represented only one hospital setting. From a strict scientific view, our findings cannot be applied to other hospital contexts. Originally, we planned to evaluate the RAFAELA system at two different hospitals in Norway. Due to administrative and economic reasons, the system was evaluated at only one hospital. It is likely to believe that participation from two or more hospital settings would have increased the external validity of our study (Shenton, 2004). Furthermore, the study was performed shortly after implementing the RAFAELA system and the informants could only refer to 0.5–2 years of RAFAELA experiences. It is possible that a longer experience of using the system would provide multiple nuances and variations to the results. Shenton proposed that the understanding of a phenomenon is gained gradually and through several studies (Shenton, 2004). Unfortunately, a long‐term perspective was not part of our study. We therefore propose further research on long‐term experiences using the RAFAELA system. Finally, respondents participating in focus group interviews in general may feel peer pressure to give similar answers to the researchers’ questions. We tried to minimize this bias by thorough information before start of the interviews and by two researchers (BML and KL) participating in the interviews to ensure integrity of participants. Depending of the power relationship between the researchers and the informants, a careful invitation to individual comments on the summary of the interviews might have revealed experiences not discussed in the focus groups.

The strength of our study lies in the representativeness of the informants. As many as 12 informants representing all units at our hospital using the RAFAELA system participated in the study. Hence, our sampling was total concerning organization level. Furthermore, by conducting focus group interviews, we stimulated reflections and broad discussions about the nurse managers’ perception.

## 5 CONCLUSION

Through focus group interviews we explored nurse managers’ perception of the RAFAELA system as used in a Norwegian hospital setting. The informants experienced the system to be a basis for a precise, common language and contributing to define quality standards of nursing care. Though RAFAELA was considered time‐consuming, the system provided daily documentation of nursing intensity and thus was considered an important management tool for balancing patient needs with appropriate staff. Our study provides new insight about the RAFAELA system with the perspective of nurse managers. We conclude that nurse managers at medical and surgical hospital units can benefit from data that the system generates.

## CONFLICTS OF INTEREST

The authors declare no conflict of interests.

## References

[nop2115-bib-0001] Aiken, L. H. , Cimiotti, J. P. , Sloane, D. M. , Smith, H. L. , Flynn, L. , & Neff, D. F. (2011). Effects of nurse staffing and nurse education on patient deaths in hospitals with different nurse work environments. Medical Care, 49(12), 1047–1053. https://doi.org/10.1097/MLR.0b013e3182330b6e 21945978 10.1097/MLR.0b013e3182330b6e PMC3217062

[nop2115-bib-0002] Aiken, L. H. , Sloane, D. M. , Bruyneel, L. , van den Heede, K. , Griffiths, P. , Busse, R. , & Sermeus, W. (2014). Nurse staffing and education and hospital mortality in nine European countries: A retrospective observational study. Lancet, 383(9931), 1824–1830. https://doi.org/10.1016/s0140-6736(13)62631-8 24581683 10.1016/S0140-6736(13)62631-8 PMC4035380

[nop2115-bib-0003] Andersen, M. H. , Lønning, K. , Bjørnelv, G. M. , & Fagerstrom, L. (2016). Nursing intensity and costs of nurse staffing demonstrated by the RAFAELA system: Liver vs. kidney transplant recipients. Journal of Nursing Management, 24(6), 798–805. https://doi.org/10.1111/jonm.12384 27162168 10.1111/jonm.12384

[nop2115-bib-0004] Andersen, M. , Lønning, K. , & Fagerström, L. (2014). Testing reliability and validity of the oulu patient classification instrument—The first step in evaluating the RAFAELA system in Norway. Open Journal of Nursing, 4, 303–311. https://doi.org/10.4236/ojn.2014.44035

[nop2115-bib-0005] Andreasson, J. , Eriksson, A. , & Dellve, L. (2015). Health care managers’ views on and approaches to implementing models for improving care processes. Journal of Nursing Management, 24, 219–227. https://doi.org/10.1111/jonm.12303 25851462 10.1111/jonm.12303

[nop2115-bib-0006] Baernholdt, M. , & Cottingham, S. (2011). The clinical nurse leader–New nursing role with global implications. International Nursing Review, 58(1), 74–78. https://doi.org/10.1111/j.1466-7657.2010.00835.x 21281297 10.1111/j.1466-7657.2010.00835.x

[nop2115-bib-0007] Brown, D. S. , Donaldson, N. , Burnes Bolton, L. , & Aydin, C. E. (2010). Nursing‐sensitive benchmarks for hospitals to gauge high‐reliability performance. Journal for Healthcare Quality, 32(6), 9–17. https://doi.org/10.1111/j.1945-1474.2010.00083.x 10.1111/j.1945-1474.2010.00083.x 20946421

[nop2115-bib-0008] Fagerström, L. , Lønning, K. , & Andersen, M. H. (2014). The RAFAELA system: A workforce planning tool for nurse staffing and human resource management. Nursing Management (Harrow, London, England: 1994), 21(2), 30–36. https://doi.org/10.7748/nm2014.04.21.2.30.e1199 10.7748/nm2014.04.21.2.30.e1199 24779764

[nop2115-bib-0009] Fagerström, L. , Rainio, A. K. , Rauhala, A. , & Nojonen, K. (2000). Validation of a new method for patient classification, the oulu patient classification. Journal of Advanced Nursing, 31(2), 481–490. https://doi.org/10.1046/j.1365-2648.2000.01277.x 10672108 10.1046/j.1365-2648.2000.01277.x

[nop2115-bib-0010] Fagerström, L. , & Rauhala, A. (2007). Benchmarking in nursing care by the RAFAELA patient classification system ‐ a possibility for nurse managers. Journal of Nursing Management, 15(7), 683–692. https://doi.org/10.1111/j.1365-2934.2006.00728.x 17897144 10.1111/j.1365-2934.2006.00728.x

[nop2115-bib-0011] Fagerström, L. , & Vainikainen, P. (2014). Nurses’ experiences of nonpatient factors that affect nursing workload: A study of the PAONCIL instrument's nonpatient factors. Nursing Research and Practice, 2014, 167674 https://doi.org/10.1155/2014/167674 25050179 10.1155/2014/167674 PMC4090478

[nop2115-bib-0012] Fasoli, D. R. , & Haddock, K. S. (2010). Results of an integrative review of patient classification systems. Annual Review of Nursing Research, 28, 295–316. https://doi.org/10.1891/0739-6686.28.295 10.1891/0739-6686.28.295 21639031

[nop2115-bib-0013] Frilund, M. , & Fagerström, L. (2009). Validity and reliability testing of the Oulu patient classification: Instrument within primary health care for the older people. International Journal of Older People Nursing, 4(4), 280–287. https://doi.org/10.1111/j.1748-3743.2009.00175.x 20925853 10.1111/j.1748-3743.2009.00175.x

[nop2115-bib-0014] Harper, K. , & McCully, C. (2007). Acuity systems dialogue and patient classification system essentials. Nursing Administration Quarterly, 31(4), 284–299. https://doi.org/10.1097/01.NAQ.0000290426.41690.cb 17909428 10.1097/01.NAQ.0000290426.41690.cb

[nop2115-bib-0015] Hustad, N. B. , Hellesø, R. , & Andersen, M. H. (2015). A qualitative study of manager experiences using the RAFAELA system. Open Journal of Nursing, 5, 1024–1032. https://doi.org/10.4236/ojn.2015.511109

[nop2115-bib-0016] Keogh, B. (2013). Review into the quality of care and treatment provided by 14 hospital trusts in England: overview report. Retrieved from https://www.nhs.uk/nhsengland/bruce-keogh-review/documents/outcomes/keogh-review-final-report.pdf.

[nop2115-bib-0017] Krueger, R. A. , & Casey, M. A. (2009). Focus groups: A practical guide for applied research, (4th edn). Los Angeles, Calif: Sage.

[nop2115-bib-0018] Kvale, S. , & Brinkmann, S. (2009). Interviews: Learning the craft of qualitative research interviewing, (2nd edn). Los Angeles, Calif: Sage.

[nop2115-bib-0019] Lang, T. A. , Hodge, M. , Olson, V. , Romano, P. S. , & Kravitz, R. L. (2004). Nurse‐patient ratios: A systematic review on the effects of nurse staffing on patient, nurse employee and hospital outcomes. Journal of Nursing Administration, 34(7–8), 326–337. https://doi.org/10.1097/00005110-200407000-00005 15303051 10.1097/00005110-200407000-00005

[nop2115-bib-0020] McHugh, M. D. , Berez, J. , & Small, D. S. (2013). Hospitals with higher nurse staffing had lower odds of readmissions penalties than hospitals with lower staffing. Health Affairs, 32(10), 1740–1747. https://doi.org/10.1377/hlthaff.2013.0613 24101063 10.1377/hlthaff.2013.0613 PMC4315496

[nop2115-bib-0021] Needleman, J. , Buerhaus, P. , Pankratz, V. S. , Leibson, C. L. , Stevens, S. R. , & Harris, M. (2011). Nurse staffing and inpatient hospital mortality. New England Journal of Medicine, 364(11), 1037–1045. https://doi.org/10.1056/NEJMsa1001025 21410372 10.1056/NEJMsa1001025

[nop2115-bib-0022] Needleman, J. , & Hassmiller, S. (2009). The role of nurses in improving hospital quality and efficiency: Real‐world results. Health Affairs, 28(4), w625–w633. https://doi.org/10.1377/hlthaff.28.4.w625 19525289 10.1377/hlthaff.28.4.w625

[nop2115-bib-0023] Porter, M. E. (2010). What is value in health care? New England Journal of Medicine, 363(26), 2477–2481. https://doi.org/10.1056/NEJMp1011024 21142528 10.1056/NEJMp1011024

[nop2115-bib-0024] Pusa, A. K. (2007). The right nurse in the right place. Nursing productivity and utilization of the RAFAELA patient classification system in nursing management. (PhD), Kuopio University Publications. Retrieved from http://epublications.uef.fi/pub/urn_isbn_978-951-27-0517-7/urn_isbn_978-951-27-0517-7.pdf.

[nop2115-bib-0025] Rafferty, A. M. , Clarke, S. P. , Coles, J. , Ball, J. , James, P. , McKee, M. , & Aiken, L. H. (2007). Outcomes of variation in hospital nurse staffing in English hospitals: Cross‐sectional analysis of survey data and discharge records. International Journal of Nursing Studies, 44(2), 175–182. https://doi.org/10.1016/j.ijnurstu.2006.08.003 17064706 10.1016/j.ijnurstu.2006.08.003 PMC2894580

[nop2115-bib-0026] Rauhala, A. , & Fagerström, L. (2004). Determining optimal nursing intensity: The RAFAELA method. Journal of Advanced Nursing, 45(4), 351–359. https://doi.org/10.1046/j.1365-2648.2003.02918.x 14756829 10.1046/j.1365-2648.2003.02918.x

[nop2115-bib-0027] Rice, P. L. , & Ezzy, D. (1999). Qualitative research methods: A health focus South Melbourne. Australia: Oxford University Press.

[nop2115-bib-0028] Ruland, C. M. , & Ravn, I. H. (2003). Usefulness and effects on costs and staff management of a nursing resource management information system. Journal of Nursing Management, 11(3), 208–215. https://doi.org/10.1046/j.1365-2834.2003.00381.x 12694368 10.1046/j.1365-2834.2003.00381.x

[nop2115-bib-0029] Rutherford, M. (2008). Standardized nursing language: What does it mean for nursing practice? Online Journal of Issues in Nursing, 13(1), 1–10.

[nop2115-bib-0030] Shenton, A. K. (2004). Strategies for ensuring trustworthiness in qualitative research projects. Education for Information, 22(2), 63–75. https://doi.org/10.3233/EFI-2004-22201

[nop2115-bib-0031] Spence, K. , Tarnow‐Mordi, W. , Duncan, G. , Jayasuryia, N. , Elliott, J. , King, J. , & Kite, F. (2006). Measuring nursing workload in neonatal intensive care. Journal of Nursing Management, 14(3), 227–234. https://doi.org/10.1111/j.1365-2934.2006.00609.x 16600012 10.1111/j.1365-2934.2006.00609.x

[nop2115-bib-0032] van Oostveen, C. J. , Ubbink, D. T. , & Huis in het Veld, J. G. , Bakker, P. J. , Vermeulen, H. (2014). Factors and models associated with the amount of hospital care services as demanded by hospitalized patients: A systematic review. PLoS ONE, 9(5), e98102 https://doi.org/10.1371/journal.pone.0098102 24878506 10.1371/journal.pone.0098102 PMC4039449

